# Second SVC stent treatment for tumour ingrowth

**DOI:** 10.1002/rcr2.619

**Published:** 2020-07-02

**Authors:** Yutaka Takahara, Yoko Ishige, Ikuyo Shionoya, Yuki Fujimoto, Taku Oikawa, Shiro Mizuno

**Affiliations:** ^1^ Department of Respiratory Medicine Kanazawa Medical University Uchinada Japan

**Keywords:** Additional stent, lung cancer, SVC syndrome, tumour ingrowth

## Abstract

We herein report a case of lung cancer with recurrent superior vena cava (SVC) syndrome, which was treated with additional stent placement. Our report suggests the possibility that additional SVC stent placement is an option for treatment of tumour ingrowth, even in patients with poor performance status.

## Graphical abstract

This is a case of lung cancer with recurrent superior vena cava (SVC) syndrome, which was treated with additional stent placement.

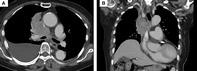

## Clinical Image

**Figure 1:**
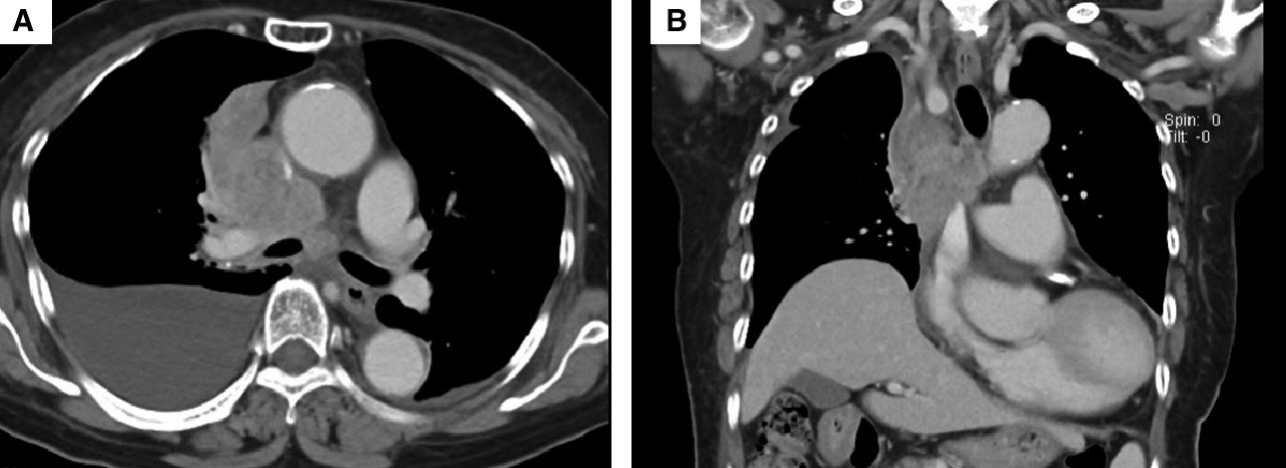
Contrast‐enhanced computed tomography (CT) images of the thorax in the axial (A) and coronal (B) planes. Chest CT showed a mediastinal mass and severe stenosis of the superior vena cava. The tumour invaded the mediastinum.

**Figure 2:**
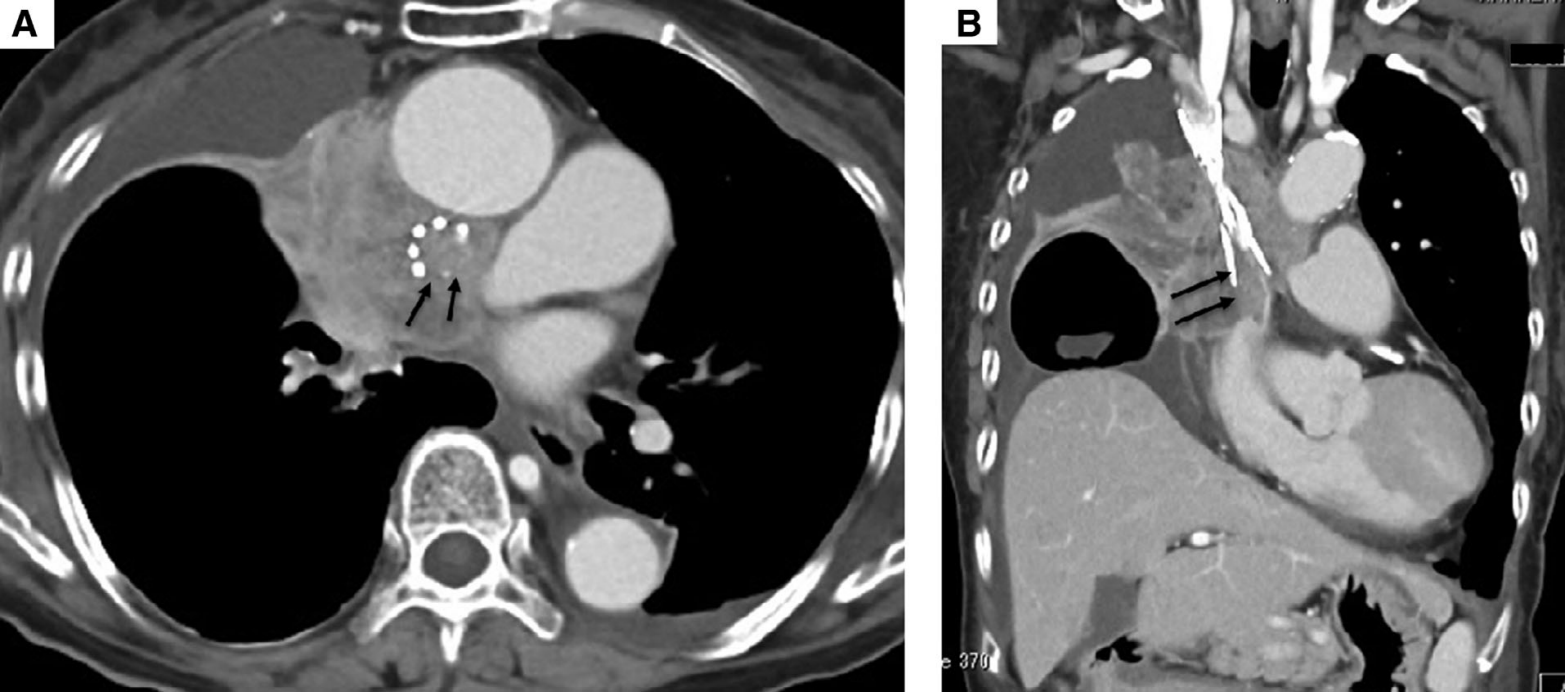
Contrast‐enhanced computed tomography (CT) images of the thorax in the axial (A) and coronal (B) planes. Chest CT showed proximal obstruction of the initial superior vena cava (SVC) stent by tumour ingrowth (arrows).

**Figure 3:**
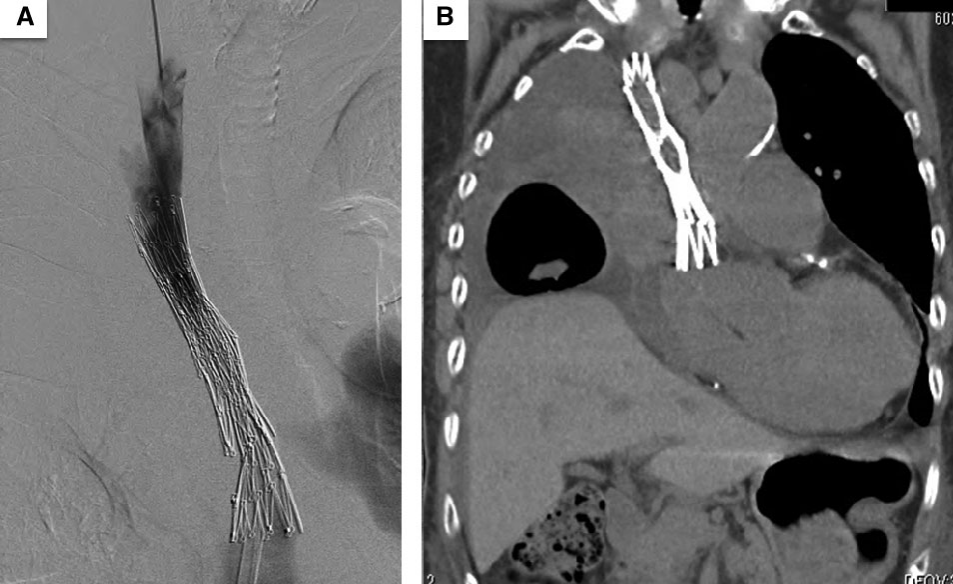
An angiograph (A) and computed tomography (CT) scan (B) of the post‐stenting superior vena cava (SVC). (A) An additional SVC stent was placed and post‐stenting venogram showed flow improvement in the SVC. (B) The distal end of the additional stent was located just proximal to the right atrium.

A 78‐year‐old woman had previously received chemoradiotherapy for lung squamous cell carcinoma. She was hospitalized with dyspnoea and oedema of the upper body. Chest computed tomography (CT) (Fig. 1) revealed worsening of the primary lesion causing superior vena cava (SVC) compression. Therefore, an SVC stent was placed. She had a performance status (PS) of 1. However, because of disease progression, her PS worsened from 1 to 3. Chest CT (Fig. 2) revealed the stent to be obstructed by tumour ingrowth. We decided to perform an additional SVC stenting. The guide wire was introduced via the right internal jugular vein. The wire was grasped with a snare on the opposite side from the right femoral vein approach. An additional SVC stent was placed, with its lower tip proximal to the right atrium (Fig. 3). After stent placement, her symptoms (dyspnoea and oedema) improved immediately with no complications. At one‐month follow‐up, she remained asymptomatic. Endovascular stents have been applied to treat SVC syndrome (SVCS). However, their effectiveness for SVCS with recurrent disease is unclear [1]. Our report suggests the possibility that an additional SVC stent is an option for treatment of tumour ingrowth, even in patients with poor PS.

### Disclosure Statement

Appropriate written informed consent was obtained for publication of this case report and accompanying images.
